# Factors affecting the scientific research ability and the corresponding countermeasures in clinical postgraduates

**DOI:** 10.1186/s12909-023-04261-w

**Published:** 2023-05-05

**Authors:** De-Qiang Fu, Yin-Qiong Huang, Yu-Hui Que, Yu Hong, Jian-Qing Lin

**Affiliations:** 1 Department of Medical Education, The Second Affiliated Hospital of Fujian Medical University, Quanzhou, 362000 China; 2 Department of Oncology, The Second Affiliated Hospital of Fujian Medical University, Quanzhou, 362000 China; 3 Department of Endocrinology, The Second Affiliated Hospital of Fujian Medical University, Quanzhou, 362000 China; 4 Thyroid & Breast Surgery, The Second Affiliated Hospital of Fujian Medical University, Quanzhou, 362000 China

**Keywords:** Clinical medicine, Postgraduate students, Scientific research ability, Investigating the status quo

## Abstract

**Background:**

Scientific research ability (SRA) is very important for clinical postgraduates. However, the factors affecting students' SRA are constantly changing with the development of medicine. The aim of this study was to investigate the current situation of SRA in clinical postgraduates and exploring the potential factors and the corresponding countermeasures under the background of new medical science.

**Methods:**

A total of 133 postgraduates (first- or second-year) were investigated by questionnaire in the Second Affiliated Hospital of Fujian Medical University. All results were analyzed by R software.

**Results:**

In terms of the SRA, academic-degree postgraduate students (ADPSs) were significantly better than professional-degree postgraduate students (PDPSs) (*P* = 0.001), the students with scientific research interest were remarkably better than those without scientific research interest (*P* = 0.004), the students who mastered statistical analysis methods were more prominent than those who did not (*P* = 0.007), the students with paper-writing skills were obviously superior to those without it (*P* = 0.003), and the second-year students were notably better than the first-year students (*P* = 0.003). Stratified analysis by the above factors except the degree type showed no significant difference in the first-year postgraduates. In the second-year postgraduates, the ADPSs were remarkably superior to the PDPSs (*P* = 0.002), the students with scientific research interest were obviously better than those without scientific research interest (*P* = 0.014), the students with more time investment in scientific research were more prominent than those with less time investment in scientific research (*P* = 0.025), the students with paper-writing skills were notably superior to those without it (*P* = 0.031), and the students with plotting ability were better than those without it (*P* = 0.013).

**Conclusion:**

The important factors affecting the SRA of clinical postgraduates include the degree type, the grade of student, scientific research interest, time investment in scientific research, statistical analysis methods, paper-writing skills, plotting ability. In short, earlier systematic SRA training contributes to the improvement of SRA in clinical postgraduates, especially in PDPSs.

**Supplementary Information:**

The online version contains supplementary material available at 10.1186/s12909-023-04261-w.

## Introduction

The purpose of medical education is to cultivate excellent medical talents [1, 2]. With the development of society, postgraduate education has become the mainstay in the medical education system [3–7]. In China, the types of master's degrees include academic degree and professional degree [8]. Academic degree is established according to disciplines and is oriented by academic research. Academic degree programs emphasize the importance of theory and research [9]. In short, the aim of academic degree is to train university teachers and researchers in scientific research institutions. Professional degree is oriented by professional practice, and professional degree programs pay more attention to practice and application [10]. The purpose of professional degree is to cultivate excellent talent who have received formal and high-level training in specialty and specialized technology [11, 12]. Thus, there is more or less a difference in scientific research ability (SRA) between the students of the two types due to different talent training mechanisms. However, the SRA plays an important role in the process of medical development [13–15].

Combining our study's understanding of the term 'SRA' with that of other studies[16–19], we provide a detailed definition as follows: SRA is a comprehensive evaluation indicator of solving scientific problems, including literature retrieval, literature review, information integration, project design, project implementation, data analysis, result verification, software application, and paper writing. SRA will promote students' innovation and continuously influence students' medical careers [16], and is also essential for the clinical doctors [20]. For basal medical researchers and clinical researchers, the project funds and the scientific research platform obviously influence their research achievements [15, 21]. For clinical postgraduates, scientific research thinking, experimental skills, statistical methods, paper-writing skills and plotting ability are regarded as the vital factors affecting SRA [22].

At present, published papers and scientific research projects are regarded as the vital quantitative indicators of SRA. In most of medical universities, the quality of postgraduate education is usually assessed according to the SRA [21]. However, parts of clinical postgraduates have to spend more time in clinical practice than in scientific research [23], their SRA will be suppressed in the long run. As we have known, the development of clinical medicine requires the promotion of scientific innovation based on the SRA [24–27]. Therefore, it is necessary to improve the SRA of clinical postgraduates through various measures.

When the training scheme for postgraduate medical education is designed, teaching hospitals need to maintain the balance between clinical practice and scientific research [28, 29], identify the related factors affecting the development of graduate education at different stages, then optimize the current training system. The aim of this study was to investigate the current situation of SRA in clinical postgraduates and exploring the potential factors and the corresponding countermeasures under the background of new medical science.

## Methods

### Study design and data collection

The observational study was based on the survey data of clinical postgraduates in China. The questionnaire survey was carried out by the Questionnaire Star platform, and the questionnaire was delivered by the WeChat platform in June 2022. It was not allowed to re-answer the questionnaire on the same IP address. The questionnaire survey was completed within 3 days, and then the survey data were collected and analyzed.

### Participants

There were about 250 postgraduates in the Second Clinical Medical College of Fujian Medical University. Considering that the timing of our investigation was at the graduation stage of the third-year postgraduates, these students were not enrolled. The remaining 170 students were invited to complete the questionnaire via Wechat. The notice was issued three times. Students who rejected to complete the questionnaire or took less than five minutes to complete it were excluded. In the end, a total of 133 students were enrolled in our study. Among them, there were 91 first-year postgraduates and 42 s-year postgraduates. All students were academic-degree postgraduate students (ADPSs) or professional-degree postgraduate students (PDPSs). The details of the students are shown in Table 1.

**Table 1:** The Characteristics of all clinical postgraduates

**Characteristics**		**n**	**Percentage(%)**
**Grade**	**First-year Grade**	**91**	**68. 42**
	**Second-year Grade**	**42**	**31.58**
**Degree Type**	**Academic Degree**	**38**	**28.57**
	**Professional Degree**	**95**	**71.43**
**Gender**	**Male**	**58**	**43.61**
	**Female**	**75**	**56.39**
**Age (years)**	**23–25**	**90**	**67.67**
	**26–30**	**38**	**28.57**
	**31–35**	**5**	**3.76**
**Professional Emphasis**	**Internal Medicine**	**39**	**29.32**
	**Surgery**	**58**	**43.61**
	**Obstetrics & Gynecology**	**3**	**2.26**
	**Paidology**	**2**	**1.50**
	**Other**	**31**	**23.30**

### Questionnaire

Our study used a self-designed Chinese questionnaire (Additional file 1) that comprised 21 (4 + 5 + 3 + 6 + 3) questions (factors). The questions included the basic information of students (native place, family, gender, occupation), profession and interest (grade, degree type, research interest, future plan, opinion on research), teacher factors (communication frequency, communication means, academic style), methods of scientific research (time investment, literature review, bioinformatics method, statistics method, paper-writing skills, plotting ability), and scientific research achievement (number of published Chinese papers, number of published SCI papers, number of projects assisted to complete). These questions were scored on a scale of Yes/positive (No/negative). The questionnaires were completed anonymously to ensure the accuracy of the data.

### Classification of SRA

All students were divided into High-SRA group and Low-SRA group according to scientific research scores. The scientific research score was calculated based on the following standard: the student who published a paper in a Chinese journal received one point; the student who published a SCI-indexed paper got two points; and the student assisted completing a project application got one point. The scores of students were the sum of their points. According to the scores, a score more than 1 was regarded as high SRA, and a score less than or equal to 1 was regarded as low SRA.

### Statistical methods

R software (version 4.1.1) was used to analyze the results. Continuous variables were expressed as the mean ± standard deviation ($\mathcal{x}$ ± s) and were analyzed by t-test. Categorical variables are expressed as percentages (%) and were analyzed by the chi-square test. *P*-values less than 0.05 were typically considered to be statistically significant.

## Results

### Factors affecting the SRA of all clinical postgraduates

**Fig. 1:**
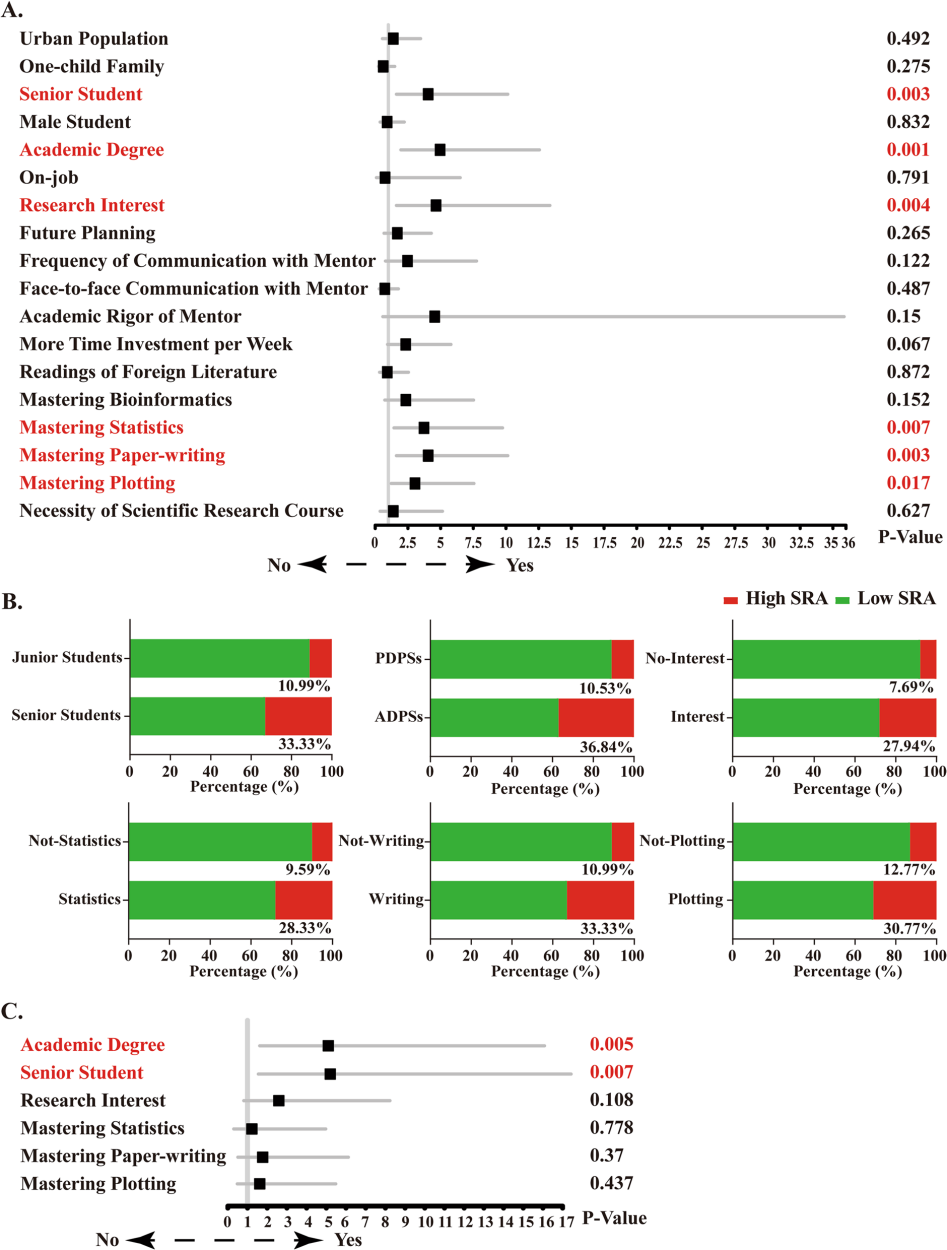
Analysis of the potential factors affecting scientific research ability in all clinical postgraduates. **A** Univariate analysis based on the potential factors; **B** The composition of SRA between different groups divided according to every core factor; **C** Multivariate analysis based on the potential factors

First, we tried to identify the potential factors affecting SRA of students based on the data of all postgraduates. The results of univariate analysis showed that 6 factors, including grade, degree type, scientific research interest, mastery of statistical methods, paper-writing skills and plotting ability, were significantly correlated with the students' SRA (Fig. 1A, Additional file 2, Supplementary Table 1). In addition, other four factors including frequency of communication with mentor, academic rigor of mentor, more time investment per week and mastery of bioinformatics, play more or less positive roles, despite no obvious correlation with SRA.

Subsequently we mapped the SRA profile of the students based on each factor. As shown in Fig. 1B, there were 14 students (33.33%) with high SRA and 28 students (66.67%) with low SRA among the second-year students and 10 students (10.99%) with high SRA and 81 students (89.01%) with low SRA among the first-year students. The senior students were significantly better than the junior students (*P* = 0.003). There were 14 students (36.84%) with high SRA and 24 students (63.16%) with low SRA in the ADPSs, 10 students (10.53%) with high SRA and 85 students (89.47%) with low SRA in the PDPSs. The ADPSs were obviously superior to the PDPSs (*P* = 0.001). There were 19 students (27.94%) with high SRA and 49 students (72.06%) with low SRA among the students with scientific research interest, 5 students (7.69%) with high SRA and 60 students (92.31%) with low SRA among the students without research interest. Students with scientific research interest were more prominent than those without scientific research interest (*P* = 0.004). There were 17 students (28.33%) with high SRA and 43 students (71.67%) with low SRA in the students mastering the statistical analysis method, 7 students (9.59%) with high SRA and 66 students (90.41%) with low SRA in the students not mastering the statistical analysis method. The students mastering the statistical analysis methods was notably better than those who did not (*P* = 0.007). There were 14 students (33.33%) with high SRA and 28 students (66.67%) with low SRA among those with paper-writing skills. There were 10 students (10.99%) with high SRA and 81 students (89.01%) with low SRA among those without paper-writing skills. The students with paper-writing skills were remarkably better than those without it (*P* = 0.003). Among the students with plotting ability, 12 students (30.77%) had high SRA, and 27 students (69.23%) had low SRA. Among the students without plotting ability, 12 (12.77%) students had high SRA, and 82 students (87.23%) had low SRA. The students with plotting ability were significantly superior to those without it (*P* = 0.017).

Further, multivariate analysis was performed to identify the core factors. Amazingly, just two factors including the grade (*P* = 0.007) and the degree type (*P* = 0.005) exhibited significant correlation with SRA (Fig. 1C, Additional file 2, Supplementary Table 2). There were no obvious differences in the other four factors. However, they also displayed a limited positive role on SRA.

### Degree type and scientific research interest were identified as the core factors associated with the SRA of senior postgraduates.

We speculated that there would be different factors on SRA in the students of different grade. Thus, stratification analysis was perform based on the grade of the students. Unexpectedly, in the first-year postgraduates, there were no significant differences between the groups divided according to each factor except the degree type (*P* = 0.044) (Additional file 2, Supplementary Table 3). Excitingly, in the second-year postgraduates, the univariate analysis showed that 5 factors, including the degree types, the scientific research interest, the time investment of scientific research, SCI paper-writing skills and plotting ability, were strongly correlated with the SRA (Table 2).

**Table 2:** Univariate analysis based on potential factors in senior clinical postgraduates

Characteristics	OR	CI5	CI95	*P*-value
Urban Population	**0.64**	**0.17**	**2.4**	**0.51**
One-child Family	**0.65**	**0.18**	**2.36**	**0.509**
Male Student	**0.75**	**0.21**	**2.73**	**0.663**
Academic Degree	**17.33**	**2.91**	**103.38**	0.002 *
On-job	**2.08**	**0.12**	**35.9**	**0.615**
Research Interest	**15**	**1.72**	**130.76**	0.014 *
Future Planning	**2.4**	**0.64**	**9.03**	**0.195**
Frequency of Communication with Mentor	**4.5**	**0.84**	**23.99**	**0.078**
Face-to-face Communication with Mentor	**0.42**	**0.11**	**1.57**	**0.195**
Academic Style of Mentor	**5.2**	**0.58**	**46.6**	**0.141**
Time Investment per Week	**4.89**	**1.22**	**19.65**	0.025 *
Readings of Foreign Literature	**0.5**	**0.09**	**2.8**	**0.431**
Mastering Bioinformatics	**1.64**	**0.31**	**8.59**	**0.561**
Mastering Statistics	**8.41**	**0.96**	**73.73**	**0.054**
Mastering Paper-writing	**4.5**	**1.15**	**17.65**	0.031 *
Mastering Plotting	**6.13**	**1.46**	**25.73**	0.013 *
Necessity of Scientific Research Course	**1.56**	**0.15**	**16.53**	**0.712**

*OR* Odds ratio, *CI5* 5% Confidence interval, *CI95* 95% Confidence interval; *P < 0.05

**Fig. 2:**
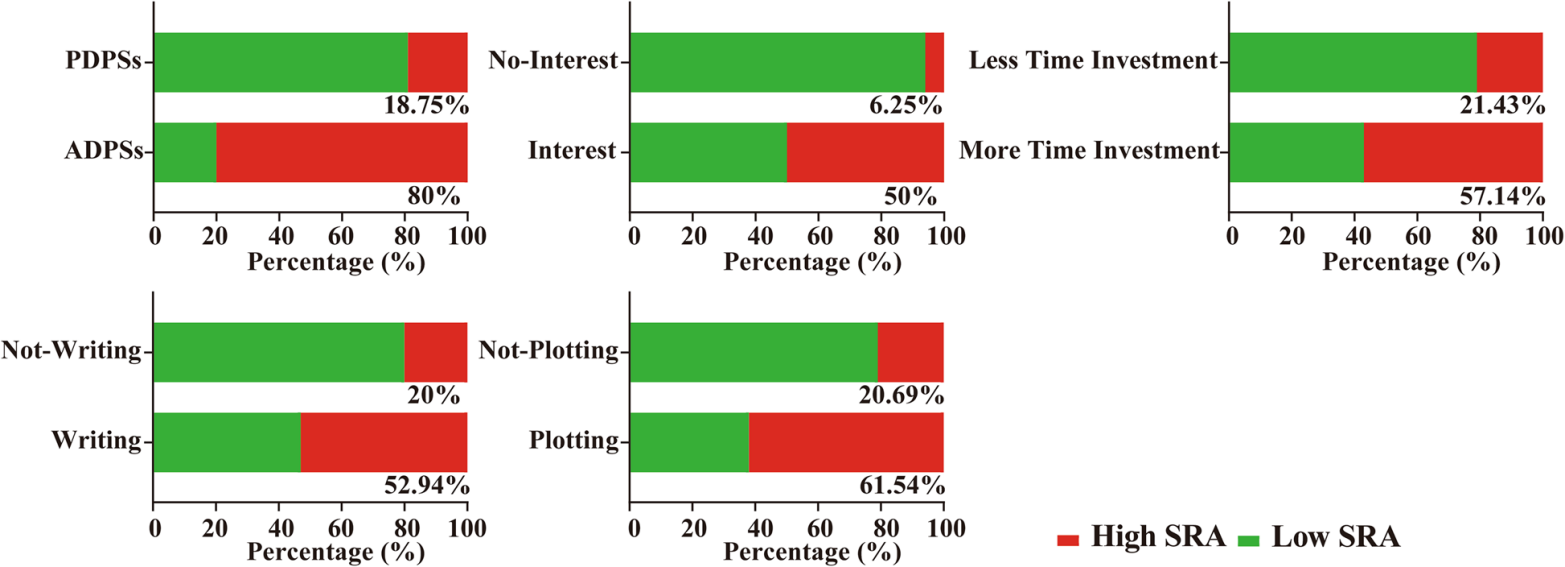
Analysis of the potential factors affecting scientific research ability in senior clinical postgraduates

Subsequently, as shown in Fig. 2, the SRA profile based on each related factor was mapped. There were 8 students (80%) with high SRA and 2 students (20%) with low SRA in the ADPSs and 6 students (18.75%) with high SRA and 26 students (81.25%) with low SRA in the PDPSs. The ADPSs were notably better than the PDPSs (*P* = 0.002). There were 13 students (50%) with high SRA and 13 students (50%) with low SRA among the students with scientific research interest, 1 student (6.25%) with high SRA and 15 students (93.75%) with low SRA among the students without scientific research interest. The students with scientific research interest were obviously superior to those without it (*P* = 0.014). There were 8 students (57.14%) with high SRA and 6 students (42.86%) with low SRA among the students with more time investment in scientific research, 6 students (21.43%) with high SRA and 22 students (78.57%) with low SRA among the students with less time investment in scientific research. The students with more time investment in scientific research were remarkably better than those without it (*P* = 0.025). There were 9 students (52.94%) with high SRA and 8 students (47.06%) with low SRA in the students with paper-writing skills, 5 students (20%) with high SRA and 20 students (80%) with low SRA in the students without it. The students with paper-writing skills were more prominent than those without it (*P* = 0.031). There were 8 students (61.54%) with high SRA and 5 students (38.46%) with low SRA among the students with plotting ability, 6 students (20.69%) with high SRA and 23 students (79.31%) with low SRA among the students without plotting ability. The students with plotting ability were obviously superior to those without it (*P* = 0.013).

Furthermore, the multivariate analysis was utilized to identify the core SRA-related factors in the second-year postgraduates. As shown in Table 3, the degree types (*P* = 0.022) and the scientific research interest (*P* = 0.045) were obviously related to the SRA. In addition, paper-writing skills and plotting ability played a certain positive role.

**Table 3:** Multivariate analysis based on potential factors in senior clinical postgraduates

Characteristics	OR	CI5	CI95	*P*-value
Academic Degree	**56.77**	**1.79**	**1802.13**	0.022 *
Research Interest	**15.72**	**1.07**	**231.43**	0.045 *
Time Investment per Week	**0.8**	**0.07**	**8.83**	**0.856**
Mastering Paper-writing	**7.03**	**0.51**	**96.58**	**0.145**
Mastering Plotting	**6.77**	**0.64**	**71.94**	**0.113**

*OR* Odds ratio, *CI5* 5% Confidence interval, *CI95* 95% Confidence interval; *P < 0.05

## Discussion

### Outstanding problems in the SRA training mode of clinical postgraduates

Scientific research is the footstone promoting the development of clinical medicine [30–32]. SRA is the basics of innovation capacity for medical researchers. Therefore, it is very important to improve students’ SRA during postgraduate medical education [33, 34]. In other words, the SRA is a solid foundation for clinical postgraduates [30]. Evidence has shown that the lack of systemic SRA training impacts their subsequent development for most clinical postgraduates [35]. In our study, the results showed that 66.67% of the second-year students were low SRA. This result suggests that most of the postgraduates have not mastered the basic methods of scientific research by the end of the second school year. The reasons are as follows. First, teaching hospitals pay more attention to clinical practice than to scientific research. Van Schravendijk et al. [36] reported that the SRA of medical students was often limited by both lack of training opportunity and the time investment for research. Second, mentors do not attach importance to the studying of their students. A systematic review considered that academic mentorship could positively impact personal development and research productivity [37]. Third, students lack much enthusiasm for scientific research. In a Germany investigation [16], scientific research was not regarded as an important part of medical career for the majority of students.

### Optimizing the system of SRA training, stimulating students' interest in scientific research, and improving students’ SRA

Based on the above, it is very important to identify the potential factors affecting the SRA of postgraduate. We found that 6 factors were greatly associated with all students' SRA, including the degree type, the grade of students, scientific research interest, statistical analysis methods, paper-writing skills and plotting ability.

At present, the degree types of medical master include academic degrees and professional degrees in China. In this study, the degree type demonstrated great correlation with the SRA in all students. This result indicates that we need to pay more attention to the PDPSs, and the training plan of the PDPSs needs to be optimized. Hart et al. [22] reported that successful completion of postgraduates' research projects required sufficient time investment. A survey of three Canadian medical schools [38] supported that too little time investment was a hindrance to scientific research. We found the similar phenomenon in the second-year postgraduates. In the current training plan of Chinese clinical postgraduate, lots of clinical practice seriously impact PDPSs' SRA training. However, ADPSs take part in less clinical practice, so they can spend sufficient time on scientific research. It may be the reason the SRA of ADPSs is superior to that of PDPSs in the postgraduates. Teaching hospitals should make a reasonable balance between clinical practice and scientific research to ensure students’ comprehensive literacy.

In our study, we found that the grade of students demonstrated important roles according to the results of multivariate analysis. Ribeiro et al.’s study [39] revealed that senior students exhibited a better ability of English application and writing skills. It was reasonable that the second-year students were better than the first-year students as far as the SRA was concerned. However, 89.01% of first-year students and 66.67% of second-year students were low SRA. In particular, 81.25% of the PDPSs were still low SRA at the end of the second school year. Interestingly, in Germany medical colleges, only 13.9% of doctoral students claimed to be working on their projects, but they did not claim to have gained SRA [16]. This indicates that it is very common to place more emphasis on clinical practice than on scientific research in the current training system of clinical postgraduates. In other words, the clinical postgraduates, especially the PDPSs, need to receive more scientific research training to further improve their SRA, and it is necessary to optimize the current training system of scientific research.

Interest is the biggest motivation of scientific research and strongly correlates with scientific research achievements [40]. Students with much enthusiasm for scientific research actively learn scientific research methods and greatly improve their SRA. In our study, scientific research interest played an important role in the second-year postgraduates. However, scientific research interest is not a core factor affecting student’s SRA in the first-year postgraduates. We noticed that the basic skills of scientific research, such as mastery of statistical methods, paper-writing skills and plotting ability, played more or less positive roles on the SRA, and exhibited a certain positive correlation with scientific research interest. As we have known, most of the first-year postgraduates just begin to learn the basic skills of scientific research in China. Thus, we speculated that students would have scientific research interest only after they had mastered the basic skills of scientific research. In a word, the basic skills of scientific research were the prerequisite of high SRA.

Interestingly, the stratified analysis showed that there was no significant correlation between the SRA and all potential factors except the degree type in first-year postgraduates. We speculate that the dominant reason is that the first-year students are at the transition between undergraduate and postgraduate levels [41]. However, it could not be accepted that 89.01% of postgraduates were still low SRA at the end of the first school year. Therefore, it was urgent to carry out various SRA trainings as early as possible during the first school year [42]. In many studies [43, 44], mentors were always considered as an important factor on the growth of postgraduates. The student-mentor relationship is largely dependent on how reliant the student is on their mentor[45].Surprisingly, we did not find a great association between the mentors and their students. From another perspective, it suggests that students' independence and self-learning ability are very important to their SRA.

### Carrying out appropriate systematic SRA training as early as possible according to student’s degree types

In recent years, many scholars [16, 18, 36, 39, 42, 46] appealed that the SRA training should be carried out as early as possible to improve student’s SRA. However, the detailed implementation plan has not been proposed. In our opinions, the appropriate course would be arranged according to student’s degree types. The details are described as follows. First, it is best to carry out SRA training at the start of the first school year. Second, increasing the opportunities of experimental operations. Third, improving reading ability of literature and writing ability. Fourth, setting integrated courses involving with software applications of scientific research, medical database applications and basic experimental operations. Fifth, the students selectively master the experimental operations according to the degree types. The training process needs to be scientifically and systematically arranged. Therefore, we define this process as systematic SRA training.

The training system of SRA is the core of medical college [47–49]. By optimizing the training system of SRA, students' interest would be grown, their subjective initiative in scientific research would be exerted, their experimental skills would be improved, and their professional horizon would be broadened [50]. Thus, the students would acquire more scientific research achievements as their SRA improved. In short, in the era of new medical science, the concept of medical education requires constant innovation with the development of society. The more analysis, summary, and attempt in teaching–learning process, the more improvement of educational quality [51]. Thus, we will cultivate an increasing amount of medical talent with high comprehensive literacy for society.

## Limitations

First, our clinical medical college is affiliated to Fujian medical university. Thus, we are only able to recruit a small sample in this study. It is a great pity that the third-year postgraduates are not enrolled in the study. In addition, the evaluation standard of students' SRA is set based on the current situation of our college. Therefore, we cannot generalize our conclusion to the entire medical education situation in China. Second, since the investigation is voluntary, participants may have rather been those with a higher interest in this topic. Thus, there may be a certain selection bias in our result. However, most of the survey items focus on the common problems affecting student's SRA. These results may also be transferrable to other university to a certain extent.

## Conclusions

This study suggests that the current situation of SRA is not optimistic in Chinese clinical postgraduates, especially in PDPSs. The innovative motivation of students will be suppressed. The factors affecting SRA include the degree type, scientific research interest, the grade of student, time investment in scientific research, statistical analysis methods, paper-writing skills, plotting ability, especially the first two. We suggest that it is necessary to carry out systematic SRA trainings as early as possible during the first school year of clinical postgraduates.

## Prospect

In our upcoming study, we will cooperate with several clinical medical schools and enroll the third-year postgraduates to increase the sample size. Furthermore, based on the findings of this study, we intend to establish corresponding systematic SRA training courses for ADPSs and PDPSs respectively. Additionally, a follow-up study should be conducted to compare the students' SRA before and after the implementation of the curriculum. Through these efforts, we aim to identify new directions for educational reform and continually optimize the training mechanism for clinical medical postgraduates. Ultimately, our goal is to cultivate a greater number of highly skilled medical professionals who can effectively meet the demands of contemporary society.

## Supplementary Information

**Additional file 1.** Questionnaire for Scientific Research Ability.

**Additional file 2: Supplementary Table 1.** Univariate analysis based on the potential factors in all medical postgraduates. **Supplementary Table 2.** Multivariate analysis based on the potential factors in all medical postgraduates. **Supplementary Table 3.** Univariate analysis based on the potential factors in the junior medical postgraduates.

## Data Availability

The datasets generated and/or analyzed during the current study are not publicly available due to limitations of ethical approval involving the participants’ data and anonymity but are available from the corresponding author on reasonable request.

## Abbreviations

SRA: Scientific Research Ability
ADPSs: Academic Degree Postgraduate Students
PDPSs: Professional Degree Postgraduate Students
