# Supplementary material for: Factors affecting the scientific research ability and the corresponding countermeasures in clinical postgraduates
Source: BMC Med Educ. 2023 May 5;23:309. doi: 10.1186/s12909-023-04261-w (PMC10161631; doi:10.1186/s12909-023-04261-w)
Supplement: Supplementary file 1 — Additional file 1. Questionnaire for Scientific Research Ability. [file 12909_2023_4261_MOESM1_ESM.docx]

**Additional file 1. Questionnaire for Scientific Research Ability**

1. Are you the urban population?

(1) Yes (2) No

2. Do you come from one-child family?

(1) Yes (2) No

3. Are you a male student?

(1) Yes (2) No

4. Are you on job?

(1) Yes (2) No

5. Are you a senior student?

(1) Yes (2) No

6. Are you a postgraduate of academic-degree?

(1) Yes (2) No

7. Are you interest in scientific research?

(1) Yes (2) No

8. Do you plan to study for doctorate in future?

(1) Yes (2) No

9. Do you think it is necessary to set up scientific research courses?

(1) Yes (2) No

10. Do you communicate with your mentor more than three times a month?

(1) Yes (2) No

11. Do you mainly communicate with your mentor by face-to-face interaction?

(1) Yes (2) No

12. Is your mentor's academic style rigorous?

(1) Yes (2) No

13. Do you spend more than 3 days each week on scientific research?

(1) Yes (2) No

14. Do you read more than 4 articles every week?

(1) Yes (2) No

15. Have you mastered bioinformatics analysis methods?

(1) Yes (2) No

16. Have you mastered statistical analysis methods?

(1) Yes (2) No

17. Have you mastered SCI paper-writing skills?

(1) Yes (2) No

18. Have you mastered plotting methods?

(1) Yes (2) No

19. How many papers have you published in Chinese journal?

(1) Yes (2) No

20. How many papers have you published in SCI journal?

(1) Yes (2) No

21. How many projects have you assisted your mentor in completing?

(1) Yes (2) No
