# Supplementary material for: Factors affecting the scientific research ability and the corresponding countermeasures in clinical postgraduates
Source: BMC Med Educ. 2023 May 5;23:309. doi: 10.1186/s12909-023-04261-w (PMC10161631; doi:10.1186/s12909-023-04261-w)
Supplement: Supplementary file 2 — Additional file 2: Supplementary Table 1. Univariate analysis based on the potential factors in all medical postgraduates. Supplementary Table 2. Multivariate analysis based on the potential factors in all medical postgraduates. Supplementary Table 3. Univariate analysis based on the potential factors in the junior medical postgraduates. [file 12909_2023_4261_MOESM2_ESM.docx]

| **Additional file 2. Supplementary Table**  **Supplementary Table 1 Univariate analysis based on the potential factors in all medical postgraduates** | | | | |
| --- | --- | --- | --- | --- |
| **Characteristics** | **OR** | **CI5** | **CI95** | **Pvalue** |
| **Urban Population** | 1.38 | 0.55 | 3.47 | 0.492 |
| **One-child Family** | 0.61 | 0.25 | 1.49 | 0.275 |
| **Senior Student** | 4.05 | 1.62 | 10.14 | 0.003 * |
| **Male Student** | 0.91 | 0.37 | 2.22 | 0.832 |
| **Academic Degree** | 4.96 | 1.96 | 12.56 | 0.001 * |
| **On-job** | 0.75 | 0.09 | 6.5 | 0.791 |
| **Research Interest** | 4.65 | 1.62 | 13.36 | 0.004 * |
| **Future Planning** | 1.69 | 0.67 | 4.29 | 0.265 |
| **Frequency of Communication with Mentor** | 2.47 | 0.78 | 7.75 | 0.122 |
| **Face-to-face Communication with Mentor** | 0.73 | 0.3 | 1.77 | 0.487 |
| **Academic Style of Mentor** | 4.55 | 0.58 | 35.86 | 0.15 |
| **Time Investment per Week** | 2.33 | 0.94 | 5.79 | 0.067 |
| **Readings of Foreign Literature** | 0.92 | 0.33 | 2.54 | 0.872 |
| **Mastering Bioinformatics** | 2.34 | 0.73 | 7.52 | 0.152 |
| **Mastering Statistics** | 3.73 | 1.43 | 9.74 | 0.007 * |
| **Mastering Paper-writing** | 4.05 | 1.62 | 10.14 | 0.003 * |
| **Mastering Plotting** | 3.04 | 1.22 | 7.55 | 0.017 * |
| **Necessity of Scientific Research Course** | 1.38 | 0.37 | 5.14 | 0.627 |
| OR, odds ratio; CI5, 5% Confidence interval; CI95, 95% Confidence interval; * P < 0.05 | | | | |

| **Supplementary Table 2 Multivariate analysis based on the potential factors in all medical postgraduates** | | | | |
| --- | --- | --- | --- | --- |
| **Characteristics** | **OR** | **CI5** | **CI95** | **Pvalue** |
| **Academic Degree** | 5.1 | 1.62 | 16.08 | 0.005 * |
| **Senior Student** | 5.2 | 1.55 | 17.43 | 0.007 * |
| **Research Interest** | 2.59 | 0.81 | 8.23 | 0.108 |
| **Mastering Statistics** | 1.22 | 0.3 | 4.97 | 0.778 |
| **Mastering Paper-writing** | 1.77 | 0.51 | 6.12 | 0.37 |
| **Mastering Plotting** | 1.62 | 0.48 | 5.48 | 0.437 |
| OR, odds ratio; CI5, 5% Confidence interval; CI95, 95% Confidence interval; * P < 0.05 | | | | |

| **Supplementary Table 3 Univariate analysis based on the potential factors in the junior medical postgraduates** | | | | |
| --- | --- | --- | --- | --- |
| **Characteristics** | **OR** | **CI5** | **CI95** | **Pvalue** |
| **Urban Population** | **2.03** | **0.52** | **7.94** | **0.307** |
| **One-child Family** | **0.71** | **0.18** | **2.73** | **0.617** |
| **Male Student** | **0.92** | **0.24** | **3.52** | **0.905** |
| **Academic Degree** | **4.02** | **1.04** | **15.62** | **0.044 *** |
| **On-job** | **0** | **0** | **Inf** | **0.994** |
| **Research Interest** | **1.87** | **0.49** | **7.15** | **0.357** |
| **Future Planning** | **1.69** | **0.41** | **7** | **0.471** |
| **Frequency of Communication with Mentor** | **1.68** | **0.33** | **8.52** | **0.529** |
| **Face-to-face Communication with Mentor** | **1.69** | **0.41** | **7** | **0.471** |
| **Academic Style of Mentor** | **16288562.36** | **0** | **Inf** | **0.994** |
| **Time Investment per Week** | **1.08** | **0.26** | **4.54** | **0.916** |
| **Literature Reading** | **1.79** | **0.46** | **6.94** | **0.401** |
| **Mastering Bioinformatics** | **2.64** | **0.47** | **14.95** | **0.272** |
| **Mastering Statistics** | **1.41** | **0.37** | **5.43** | **0.617** |
| **Mastering Paper-writing** | **3.05** | **0.8** | **11.63** | **0.102** |
| **Mastering Plotting** | **1.79** | **0.46** | **6.94** | **0.401** |
| **Necessity of Scientific Research Course** | **0.91** | **0.17** | **4.72** | **0.91** |
| OR, odds ratio; CI5, 5% Confidence interval; CI95, 95% Confidence interval; * P < 0.05 | | | | |
